# Identification of enteric viruses from raw water using ﬂuoro-immuno-magnetic separation coupled to RT-PCR

**DOI:** 10.7705/biomedica.6032

**Published:** 2021-12-15

**Authors:** Raquel A. Villamizar, Dioselina Peláez-Carvajal, Luis Felipe Acero

**Affiliations:** 1 Departamento de Medicina, Facultad de Salud, Universidad de Pamplona, Pamplona, Colombia Facultad de Salud Universidad de Pamplona Pamplona Colombia; 2 Grupo de Virología, Dirección de Redes en Salud Pública, Instituto Nacional de Salud, Bogotá D.C., Colombia Instituto Nacional de Salud Bogotá D.C Colombia

**Keywords:** rotavirus infections, hepatitis A, antibodies, raw water, water purification, magnetic segregation, infecciones por rotavirus, hepatitis A, anticuerpos, agua cruda, purificación del agua, segregación magnética

## Abstract

**Introduction:**

Enteric viruses have been associated with the production of a variety of diseases transmitted by the fecal-oral route and carried through contaminated food and water. Given their structure and composition, they are highly resistant to environmental conditions and most of the chemical agents used in the purification processes. Therefore, the systematic monitoring of raw water is necessary to ensure its quality especially when it is used for producing drinking water for human consumption.

**Objective:**

We identifed the presence of rotavirus and hepatitis A virus by means of the ﬂuoro-immuno-magnetic separation technique (FIMS) in raw water taken from four purification plants and their water supplies in the department of Norte de Santander.

**Materials and methods:**

The viruses were captured and separated from the water samples using magnetic microparticles functionalized with monoclonal anti-Hepatitis A and antiRotavirus antibodies. Confocal microscopy was used to monitor the viral concentration process and transmission electron microscopy for the morphological visualization of the separated viruses. The reverse transcriptase-coupled polymerase chain reaction (RT-PCR) was applied to confrm the presence of pathogens.

**Results:**

The two enteric viruses were identifed in the majority of the analyzed water samples including water supply sources.

**Conclusion:**

We determined that the FIMS technique together with RT-PCR is highly effective for the detection of viral pathogens in complex matrices such as raw water.

## Resumen

**Introducción.:**

Los virus entéricos se asocian con una serie de enfermedades transmitidas por vía fecal-oral en alimentos o agua contaminada. Dada su estructura y composición, son muy resistentes a las condiciones ambientales y a la mayoría de los agentes químicos empleados en los procesos de potabilización, por lo cual es necesario un monitoreo sistemático del agua cruda para asegurar su calidad, máxime cuando se emplea como materia prima en la producción de agua potable para consumo humano.

**Objetivo.:**

Determinar la presencia de rotavirus y del virus de la hepatitis A mediante la técnica de separación ﬂuoro-inmuno-magnética en agua cruda procedente de cuatro plantas de potabilización y sus fuentes hídricas en el departamento de Norte de Santander.

**Materiales y métodos.:**

Los virus fueron capturados y separados a partir de las muestras de agua, empleando micropartículas magnéticas funcionalizadas con anticuerpos monoclonales anti-hepatitis A y anti-rotavirus. Se empleó microscopía confocal para hacer el seguimiento del proceso de concentración viral y, microscopía electrónica de transmisión, para la visualización morfológica de los virus separados. La reacción en cadena de la polimerasa acoplada a transcriptasa inversa (RT-PCR) se utilizó para confrmar la presencia de los patógenos.

**Resultados.:**

Los dos virus entéricos se detectaron en la mayoría de las muestras de agua analizadas, incluidas las de sus fuentes hídricas.

**Conclusión.:**

La técnica de separación ﬂuoro-inmuno-magnética acoplada a RT-PCR fue altamente efectiva en la detección de patógenos virales en matrices complejas como el agua cruda.

About 783 million people around the world lack access to drinking water and at least 2.5 billion people have no proper sanitation ^1^. In Colombia, most of the rural population faces a critical situation due to the lack of quality water. In Colombia, the physicochemical and microbiological quality of raw water is regulated by decree 1594 of 1984 ^2^; however, it is worrying that the decree does not include virological analyses.

Viruses may enter the food chain through wastewater containing excretions from infected people with contents ranging from 10^5^ to 10^11^ viral particles per gram of feces ^3^. Enteric viruses such as Enterovirus, Astrovirus, Calicivirus, Hepatitis A, and Rotavirus have devastating effects around the world, and they have been classified by the World Health Organization (WHO) from moderate to highly significant depending on the country and its socioeconomic conditions ^4^. They are usually transported by water and produce gastroenteritis, hepatitis, meningitis, or encephalitis to consumers, but they can also remain latent ^5^.

Many methods for virus detection have been reported. However, most of them require pre-concentration steps as viruses are easily diluted in water. Among the most widely used techniques, we can mention matrices based on activated carbon ^6^ or ion exchange resins ^7^, as well as absorption-elution tests, among others ^8^^,^^9^.

The immunomagnetic separation (IMS) method is effective in the detection of several enteric viruses including Hepatitis A ^10^. Recently, some of the authors in the present study reported the use of a new concentration technique called fluoro-immunomagnetic separation (FIMS) with high efficiency in the capture, concentration, and determination of Rotavirus in drinking water rendering results in just two hours ^11^. This time, we used the FIMS to detect the enteric viruses in a complex matrix such as raw water.

Surface water contaminated by sewage waste is generally used to produce drinking water around the world. Several national and international reports have detected the presence of enteric viruses in raw and drinking water ^12^^,^^13^. In this context, we report the presence of enteric viruses in raw water in the department of Norte de Santander, Colombia using FIMS and RT-PCR for the first time. Our study is in line with the provisions of the Environmental Protection Agency of the United States, which recently published a draft with a list of contaminants (CCL4) that should be monitored in waters including the Hepatitis A Virus (HAV) ^14^.

## Materials and methods

### Reagents

We used monoclonal anti-Hepatitis A (AMSBIO) and anti-Rotavirus (Millipore) antibodies at a concentration of 100 μg/ml, which were then diluted in PBS (Dulbecco, Sigma Aldrich) for a final concentration of 10 μg/mL (pH 72) and stored at -20 ◦C until use. We diluted the magnetic microparticles (1% w/v; Spherotech Inc.) in distilled water and stored them at room temperature until use. We extracted the viral RNA with a QIAamp kit (QIAGEN, Germany) and performed the RT-PCR using a SuperScript™ III One-Step RT-PCR System with Platinum™ Taq DNA Polymerase kit (Invitrogen). The primer sequence for Rotavirus and Hepatitis A amplification is shown in table 1.

**Table 1:** Primer sequences used in one-step RT-PCR for rotavirus and hepatitis A virus amplification

Name	Sequence	Product Size (Pb)
RTV	5`TTGCCACCAATTCAGAATAC 3`	211
	3`ATTTCGGACCATTTATAACC 5`	
HAV-1	5`CAGCACATCAGAAAGGTGAG 3`	192
	3`CTCCAGAATCATCTCCAAC 5`	

### Equipment

Virus separation and concentration were carried out with a manual magnetic separator (Spherotech Inc., USA). We used a FluoViewTM 1000 Olympus Confocal Microscope to obtain images of the captured viruses and an Electronic Transmission Microscope (Tecnai F20 Super Twin TMP) to confirm their presence. We applied negative staining and an 80 kW electric current to visualize their morphology. We quantified viral RNA in a NANODROP 2000 spectrometer (Thermo Scientific, USA) and performed the RT-PCR in a Veriti thermocycler (Applied Biosystems). The electrophoretic run gels were visualized in a Gel Doc XR system (BIO-RAD, USA), using a 100 bp DNA ladder (INVITROGEN).

### Description of the water treatment plants

We analyzed four water treatment plants located in two cities in the department of Norte de Santander including their primary sources (River Pamplonita and River Zulia). A code was assigned to each of them to avoid conflicts of interest with water treatment companies in the region.

*Treatment plant 1 (P1)*. In this plant, the treatment applied is the conventional one involving hydraulic operation and uptake, sedimentation, filtration, disinfection, and storage processes. The aqueduct system of the plant has two surface catchments with an average processing capacity of 110 l/sec. Its main source of water supply is the Pamplonita River.

*Treatment plant 2 (P2)*. This is an integrated plant which includes all the normal unit processes found in a conventional treatment plant space.. Its aqueduct system is supplied by two different streams, one of them from the sources of the Pamplonita River. The plant has an average processing capacity of 48 l/sec.

*Treatment plant 3 (P3)*. The purification process in this facility begins at the catchment with a lateral intake on the Pamplonita River, then the sand is extracted in four grit chambers. Flow is measured in three Parshall gutters and then conducted to a pre-sedimentation tank. The settled water is divided int two flows: the first one is directed to a Parshall gutter while the second goes to a concrete structure called a rectangular weir where the coagulant and the polymer are added. The water is flocculated, settled, and filtered in both flows and finally chlorinated and delivered to the distribution network by pumping and re-pumping.

*Treatment plant 4 (P4)*. Here, water from the Zulia River is processed by means of three large capacity electric pumps. At the plant, coagulant and polymer agents are added to the water that, once flocculated, passes through the sedimentation unit where the particles from flocculation are extracted. Next, the water goes to the filtering bed to be finally chlorinated and sent by pumping to the peripheral areas of the city it serves.

### Raw water sampling and pre-treatment

**Figure 1:**
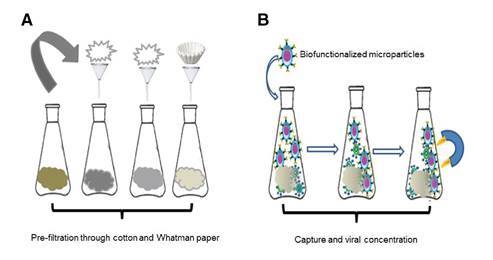
(A) Raw water pretreatment and (B) schematic representation of the magnetic concentration and separation process of rotavirus and HAV particles

We collected one liter of raw water from each treatment plant catchment and its primary source (table 2). Samples were taken by triplicate and placed into sterile portable refrigerators containing dry ice and transported to the laboratory to perform physicochemical and virological analyses. Due to the complex nature of the sample, we conducted a filtration process before the virological analyses using cotton and Whatman No.1 filter paper followed by centrifugation at 3500 rpm for 15 min (figure 1A).

**Table 2:** Geographical description of two of the most important water sources supplying water to the department of Norte de Santander, Colombia

Water source	Description
Pamplonita River	It is born in the Altogrande hill in the Páramo Fontibón, municipality of Pamplona. It flows into the Zulia River, department of Norte de Santander, Colombia. This 160 km long river is located at 8°19"47"N 72° 26"33"W, it has an average flow of 15 m^3^/s, and it drains a basin of 1,345 km^2^
Zulia River	It is born in the Páramo Cachirí at 4222 masl, department of Norte de Santander (Colombia) and is located at 9°03"23"N 72° 17"44"W. It flows into the Maracaibo Lake in Venezuela. Its length in the Colombian section is 154 km. The surface of its basin is 3,484 km^2^ with an average flow of 50 m^3^/s.

### Physicochemical analyses of raw and potable waters

We analyzed the raw water samples following the relevant parameters in Chapter IV-article 39 of decree 1594 of 1984 issued by the Ministry of Agriculture of Colombia ^2^ (table 2).

### Microparticle functionalization

We incubated a 10% microparticle solution with a 10 ppm solution of antiHepatitis A and anti-Rotavirus antibodies for 1 h at 37 °C; then we washed the microparticles twice in a 0.15 mM PBS solution containing 0.1% BSA and finally washed them again just once with distilled water. The functionalized microparticles were stored at 4 °C until use ^11^.

### Detection of Hepatitis A and Rotavirus in raw water samples

We added 500 μΙ of functionalized microparticles with anti-Hepatitis A and anti-Rotavirus antibodies to 1 L of pre-treated raw water. The particles were kept in constant agitation for 2 h at room temperature for immunoreaction. We then applied an external magnetic field to concentrate and separate the microparticle-antibody-virus complex (figure 1B). We stored the magnetized sample in an Eppendorf tube for microscopic and molecular characterization. The procedure was done in triplicate ^11^.

### Molecular characterization

*Viral RNA extraction*. The antigen-antibody interaction was broken by heating at 95 °C for five minutes and further cooling at 4 °C. Next, the microparticles were centrifuged at 10,000 rpm for two minutes ^13^. The supernatant was used to extract the viral RNA using a Qiagen Kit following the manufacturer’s instructions. Then, we treated 140 μl of the supernatant to obtain 60 μl of the final RNA extract. The amount of nucleic acid was determined by spectrophotometry ^11^.

*RT-PCR*. We amplified the viral genetic material using one-step RT- PCR. To obtain a final reaction volume of 25 μl, we added the following components: 12.5 μl of 2X PCR buffer, 1 μl of each primer at 10 μΜ, 1 μl of SuperScript™ III one-step RT-PCR enzymes, 3 μl of the RNA template, and 6.5 μl of reagent grade water. For reverse transcription, we used a 30 min cycle at 55 °C followed by a 2 min cycle at 94 °C. Denaturation was done at 94 °C for 3 min. We run the PCR through 35 cycles as follows: 94 °C for 30 s, 55 °C for 30 s, and 68 °C for 1 min. Finally, we added a 5 min extension cycle at 68 °C. Electrophoresis was run at 90 volts for 45 min in a 1.8% agarose gel in 1X TAE. We used BlueJuice™ (Invitrogen) loading buffer containing 1 μl of the molecular marker. The gel was stained with SYBR® Green for the visualization of the DNA bands. The corresponding images were documented in BioRad Gel Doc XR equipment. The amplified products were 192 bp for HAV and 211 bp for Rotavirus (table 1).

## Results

Table 3 shows the parameters and analysis methods used and table 4 the physicochemical analyses done on the samples from the catchments of each of the four water purification plants including their primary sources, the Pamplonita and Zulia Rivers.

**Table 3:** Physicochemical parameters analyzed in raw waters

Parameter	Analysis method
Real color	Spectrophotometric
pH	Electrometric
Temperature	Electrometric
Turbidity	Nephelometric
Conductivity	Electrometric
Alkalinity	Volumetric
Total hardness	Volumetric

**Table 4:** Physicochemical analysis of raw water samples from the department of Norte de Santander (Colombia).

Analysis parameter	Primary source		Catchment				Maximum permitted Decree 1594/84
	Pamplonita River	ZuliaRiver	P1	P2	P3	P4	
Color	1990	483	77	24	76	45	20 UPC
pH	7.31	7.29	7.47	7.73	7.46	7.28	6.5-8.5 units
Temperature	22	24	10	8	25	23	20° C (Optimal)
Turbidity	976	373	10.5	7.17	389	267	10 NTU
Conductivity	276	97.5	68.6	34.9	201	96.5	< 1,000 microsiemens/cm
Alkalinity	139	47	24	21	62	68	200 mg/l CaCO3
Total hardness	28	17	14	12	22	23	300 mg/l CaCO3

Results were compared to those proposed by the standards methods estipulated in Colombian regulations. Conductivity, pH, alkalinity, and total hardness were in the accepted ranges according to Decree 1594 ^2^, as previously reported by García, *et al*. ^15^. The temperature was found to vary according to the altitude but it complied with the ranges established in the decree. However, the color significantly exceeded the norm possibly explained by the dragging of decomposing sand, clay, and biological material aggregates dissolving mineral compounds in the river waters, which affects their color. In the case of turbidity, all treatment plants, except P-2, were found to surpass the established limits. Such turbidity originates from the small suspended colloidal particles dragged along the basins that reach the catchments of the water treatment plants.

**Figure 2:**
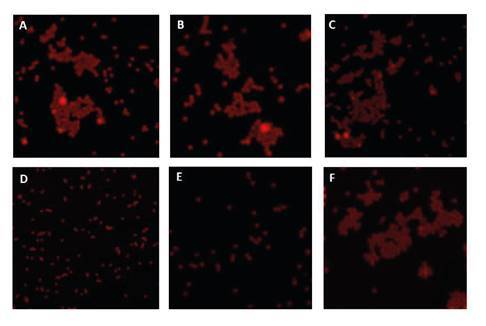
Confocal microscopy of viral complexes obtained from water samples in four potabilization plants in the department of Norte de Santander (Colombia) and their primary water sources (A: P1, B: P2, C: P3, D: P4; E, and F: Pamplonita River and Zulia River) with potential HAV content. The formation of aggregates can be observed in A, B, C, and F, while it is absent in D and E. Similar behavior was seen for Rotavirus.

We determined the presence of enteric viruses using confocal microscopy. We detected the formation of small clusters in some of the samples (figure 2). For the positive control of these assays, we used artificially spiked water with Rotavirus particles.

**Figure 3:**
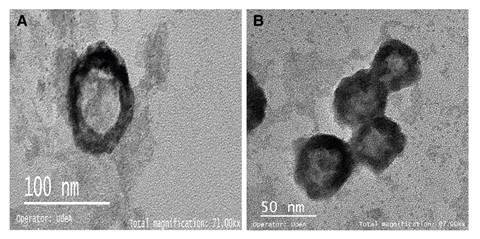
Captured and concentrated virions of A) Rotavirus and B) HAV observed by electron transmission microscopy

To confirm that clusters were induced by viruses, we analyzed by TEM. Previously, the antigen-antibody interaction was broken by applying thermal shock to facilitate the release of the viral particles. Figure 3 A  shows a TEM image of Rotavirus ranging from 50 to 80 nm diameter due to the protein coat that can sometimes be double or triple ^16^. Figure 3 B  shows the morphological features of the Hepatitis A virus similar to other enteroviruses: rounded, not wrapped, and about 30 nm diameter ^17^.

**Figure 4:**
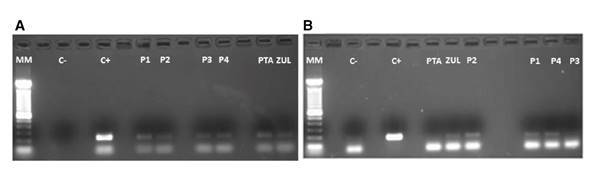
Agarose gel electrophoresis (1.8%) where C+ in A) shows the+211 bp-positive control corresponding to the VP6 gene that encodes the VP6 capsid protein while in B) C+ shows the 192 bp positive control corresponding to a fragment of the capsid protein precursor region which generates a unique intermediate (VP1-2A) of the HAV. The subsequent columns in both images show the analyzed water samples in the different treatment plants (P1-P4) and their primary water source (PTA: Pamplonita; ZUL: Zulia).

The virions released from each sample were molecularly characterized by RT-PCR. The electrophoretic run in figure 4A and B confirmed the presence of RT in all raw water samples from all treatment plants including their primary sources while HAV was not detected in plant 3 nor in the Pamplonita River.

## Discussion

According to the *Corporación Autónoma Regional de la Frontera Nororiental* (CORPONOR - Regional Autonomous Corporation of the Northeast Frontier), the greatest threat posed on the two main water sources in Norte de Santander (i.e., the Pamplonita and Zulia Rivers) is the dumping of non-treated wastewater. The Pamplonita River both supplies 10 municipalities and collects all their non-pre-treated wastewaters. The Zulia River, on the other hand, receives discharges from the capital of the department whose population nears 800,000 ^18^. The capital city harbors food and textile industries, tanneries, slaughterhouses, exploitation mines, and dry cleaning and laundry services generating solid waste, putrescible organic matter, and other liquid effluents containing highly polluting chemical materials.

According to our physicochemical analyses and the parameters in the Colombian regulations on drinkable water and basic sanitation (RAS, 2000) ^19^, which classify water quality according to its level of pollution, the color of the water samples was very deficient. As for their turbidity, the water from P1 and P2 plants showed an intermediate quality while that from P3 and P4 plants would classify as very poor. Our results match previous reports on different rivers along the country. The physicochemical dynamics in several segments of the Opia River in the department of Tolima reported low-to-medium water quality ^20^. Similarly, the surface waters supplying the municipality of Bahía Solano (department of Chocó) corresponded to low-intermediate quality as a result of sewage and anthropogenic solid waste discharges ^21^.

The physicochemical features of these water samples are adequate for the survival of viral particles. These pathogens are able to survive in a 3-10 pH range for up to 120 days in superficial and wastewaters at temperatures ranging from 20 to 30 °C ^22^. This undoubtedly represents a significant public health issue since these hydric sources are used for recreation (watering places and natural pools), irrigation, and even potabilization. The water samples we analyzed were classified as “very deficient” and, therefore, they require specific treatments to eliminate the presence of both chemical and biological contaminants. The treatment plants under study used conventional technologies for purification, which is not enough to prevent the transport of enteric viruses such as Rotavirus and HAV.

**Figure 5:**
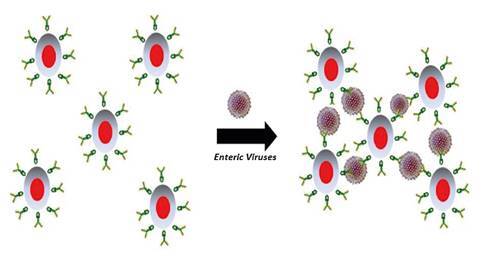
Schematic representation of enteric virus-mediated aggregate formation with fluoro- immuno-magnetic microparticles.

The virological analysis using the FIMS technique and RT-PCR succeeded in concentrating and detecting the two viral pathogens under study. The use of magnetic microparticles with a fluorescent core represents an advantage as combined, they allow for observing the viral concentration process. The formation of aggregates suggested the presence of enteric viruses in the samples (figure 2A -B-C-F). Virus-mediated clustering has been previously reported by Koh, et al., who indicate that a mono-dispersed solution of magnetic particles conjugated with antibodies is capable of producing aggregates in the presence of viral particles with binding specificity for conjugated antibodies resulting in supramolecular structures with improved magnetic properties ^23^ (figure 5).

The concentration of the enteric viruses was possible due to the complex formed between monoclonal antibodies and fluoro-magnetic microparticles. Antibodies are Y-shaped protein molecules that selectively detect structurally intact epitopes present only in infectious particles. The recognition event is mediated by supramolecular interactions based on hydrogen bonds, ionic bonds, and van der Waals forces ^24^. The Rotavirus genus is divided into seven serological groups, A to G, with several subgroups. Groups A and C include most of the human pathogens while the other groups exhibit more affinity for animals. Group A is the most common human pathogen ^17^. Here we used specific monoclonal antibodies to detect epitope VP6 in the viral capsid of group A and confirmed its presence in the catchments of the four treatment plants and their primary sources (the Pamplonita and Zulia Rivers) (figure 4A). According to the CDC, Rotavirus has a long persistence in water supplies and high infectivity ^25^ as in our findings.

In contrast, HAV was detected only in three of the four plants (P1, P2, and P4) and in the waters from the Zulia River, which supplies P4 (figure 4B). This agrees with the formation of aggregates observed by confocal microscopy (figure 2). This is a highly contagious virus with a low infective dose exhibiting tropism for the epithelial cells of the digestive tract from where it travels through the bloodstream to the liver, the target organ in infectious hepatitis ^16^.

Our findings match with those from a previous report on the high prevalence of Rotavirus and HAV in samples of raw and drinking water from different municipalities in Colombia ^12^ and another study on drinking water at the distribution sites from the same four treatment plants which determined the presence of Rotavirus in P1 ^11^. This clearly indicates that water disinfection treatments are not efficient enough when it comes to removing this type of enteric virus. The water under analysis is transporting viral pathogens which could cause public health problems. The same was reported by Lodder, et al. in surface waters from 10 different locations in the Netherlands where they found that this type of water source used to produce drinking water is likely to contain a high amount of human viral pathogens, among which they highlighted the presence of Rotavirus and Norovirus ^22^.

In our study, we identified the presence of Rotavirus and HAV in raw water samples from four treatment plants of the department of Norte de Santander (Colombia) and their primary water sources. The fluoro-immuno- magnetic separation (FIMS) technique, which combines the use of antibodies and magnetic microparticles allowed us to efficiently capture, concentrate, and separate the enteric viruses from low water volumes in little time and with no energy consumption. By performing the FIMS together with RT-PCR, we confirmed the presence of the pathogens with high sensitivity and specificity. Thus, the study serves as proof of concept for enteric virus determination using an easily operable method for monitoring virological water quality at regional and national laboratories.
